# Assessment of Total Mercury in Hair, Urine and Fingernails of Small–Scale Gold Miners in the Amansie West District, Ghana

**DOI:** 10.5696/2156-9614-9.21.190306

**Published:** 2019-03-14

**Authors:** Edward Ebow Kwaansa-Ansah, Edward Kwaku Armah, Francis Opoku

**Affiliations:** Department of Chemistry, Kwame Nkrumah University of Science and Technology, Kumasi, Ghana

**Keywords:** artisanal gold miners, hair, total mercury, occupational exposure, urine

## Abstract

**Background.:**

Mercury (Hg) is a heavy metal that can cause several adverse health effects based on its form (organic, inorganic or elemental), duration and pathway of exposure. Measurement of mercury present in human biological media is often used to assess human exposure to mercury at mining sites.

**Objectives.:**

The aim of the present study was to measure the concentrations of total mercury in urine, hair, and fingernails of miners and inhabitants of Amansie West District, Ghana.

**Methods.:**

Concentrations of total mercury were measured in sixty–eight miners and twelve non–miners in the study area using cold vapor atomic absorption spectrophotometry with an automatic mercury analyzer (HG 5000).

**Results.:**

Total mercury in nails and hair of smelter miners was 3.32 ± 0.36 and 6.59 ± 0.01 μg/g, respectively. Total mercury concentrations in hair samples obtained from smelter miners were above the 1 μg/g guideline set by the United States Environmental Protection Agency (USEPA). Moreover, the total mercury concentration in urine samples was 6.97 ± 0.06 μg/L, far below the >25 μg/L level considered to be a high level of mercury contamination. The total mercury accrued by the individuals was not dependent on age, but was positively associated with duration of stay.

**Conclusions.:**

Based on the total mercury (THg) levels analyzed in the biological media, artisanal gold mining activities in Amansie West District are on the increase with a potential risk of developing chronic effects. However, the majority of the population, particularly those engaged in artisanal small–scale gold mining, are unmindful of the hazards posed by the use of mercury in mining operations. The results showed that THg in urine, hair, and fingernails more efficiently distinguished mercury exposure in people close to mining and Hg pollution sources than in people living far from the mining sites. Further education on cleaner artisanal gold mining processes could help to minimize the impact of mercury use and exposure on human health and the environment.

**Participant Consent.:**

Obtained

**Ethics Approval.:**

This study was approved by the Ghana Environmental Protection Agency and the Ministry of Local Government and Rural Development in Manso Nkwanta.

**Competing Interests.:**

The authors declare no competing financial interests.

## Introduction

Mercury (Hg) is a heavy metal with a large environmental effect on ecosystems owing to its high toxicity and ability to bioaccumulate in the aquatic food chain.^1^ Humans mainly accumulate organic Hg through the ingestion of aquatic food species, whereas exposure to elemental or inorganic Hg occurs mainly through the inhalation of gaseous Hg.^2^ Mercury can cause several adverse health effects based on its form (organic, inorganic or elemental), duration and pathway of exposure. Chronic exposure to low levels of inorganic or elemental Hg can cause behavioral and neurocognitive disturbances, kidney dysfunction and tremors.^2^ Mercury can also cause impaired hearing and vision, as well as impairment to the central nervous system.^3^

According to the United Nations Industrial Development Organization (UNIDO), at least 100 million people in Africa, Asia and South America rely on artisanal small–scale gold mining (ASGM) directly or indirectly for their livelihood.^4–8^ In addition to cocoa, which is the main export commodity, gold mining has played a vital part in the socio–economic growth of Ghana over the past decades, and the export of gold has been an important source of revenue.^9^ However, there are health concerns in mining communities due to lack of personal protective equipment for mining, unsanitary working conditions and exposure to noise, dust and Hg.^10^

The use of Hg in ASGM has received global attention as it is hazardous to human health. Most ASGM activities are performed in rivers and along river banks, as these areas have higher gold concentrations.^11^ For the extraction of gold from ore, most artisanal miners use elemental Hg to amalgamate gold, as the process remains the cheapest and most convenient technique. During amalgamation, the resulting amalgam is smelted, and Hg vapor is released and inhaled by miners directly involved in this activity and those within the vicinity, since no ventilation or respiratory protection to prevent the release of Hg vapor has been put in place.^3^ In addition, Hg can enter into the surrounding aquatic ecosystem and bioaccumulate by bacteria into methylmercury in the food chain.^12^

In Ghana, the major environmental concerns of ASGM activities are Hg contamination from environmental degradation, ecosystem physical destruction and gold processing.^13^ Small–scale miners in most of the mining communities in Ghana handle Hg without the use of effective personal protective equipment and those involved in ASGM may breathe in high concentrations of Hg, which is steadily absorbed into their bloodstream.^10^ Exposure to Hg vapor is usually linked with occupational and accidental exposures and high doses can lead to acute and chronic effects.^14^ Total mercury (THg) contents in urine, blood, and hair can be used to assess human exposure. Because sampling of human hair is non–invasive, THg levels in hair are commonly used as a bioindicator of Hg pollution compared to urine and blood.^15^,^16^ Fingernails have also been used as a bioindicator of organic Hg contamination and have been used to assess amalgam exposure by dentists as well.^17^

Mining, regardless of the mode of operation and process, has adverse effects on the environment and atmosphere. The extent of damage depends largely on the mining processing procedures being employed. In Ghana, several studies have revealed environmental concerns, including pollution and land degradation associated with mining activities. In particular, large–scale mining activities continue to reduce the vegetation of mining towns, rendering the land unsupportive for crop growth and toxic to biological diversity.^18^,^19^ In addition to land degradation, chemical contamination from the gold extraction process poses health risks for mining communities and people residing in close proximity to these sites. Small–scale mining operations involving ore processing generate dust capable of causing dust–related diseases when the generated particles fall within the respirable dust range.^20^ Furthermore, open air burning of gold amalgam produces mercury fumes, which are released into the atmosphere. This exposes miners and nearby residents to the dangers of mercury contamination.^20^ Rivers and streams are polluted by mercury suspension and unstable piles of waste, which are usually discharged into nearby water bodies during the amalgamation and sluicing process. This, in turn, leads to coloration and siltation of nearby water bodies, rendering rivers and streams unusable for both industrial and domestic purposes. Drainage of oils and lubricants into streams also causes deoxygenation of water, which threatens aquatic life. For example, Ankobra, Pra, Densu and Birim rivers, which serve communities along the watersheds, have been contaminated, as they have been turned into reservoirs for dangerous chemical disposal.^21–23^ Moreover, pits dug during the mining process pose a hazard to the community and are breeding grounds for mosquitoes with potential dangers to human health.^24^

**Abbreviations**
*ASGM*: Artisanal small–scale gold mining
*THg*: Total mercury

Individuals involved in small–scale gold mining activities are exposed to increased levels of Hg vapor, since protective and preventive measures are not often used in mining areas. Protective measures such as fume hoods can be used to decrease mercury emissions and fumes. Moreover, techniques such as chemical leaching, direct smelting and gravity have been used to eliminate Hg used in gold–mining areas.^25^ However, small–scale miners cannot afford these techniques as they require innovation, monitoring, training and capital. Even though small–scale mining contributes significantly to the Ghanaian economy, a lack of environmental awareness, resources and training among the artisanal miners has led to health hazards for the population and environmental damage to the mining communities.^26^,^27^ Thus, the majority of the population, particularly those engaged in artisanal small–scale gold mining, are unmindful of the hazards posed by the use of mercury in mining operations. The full effect of gold mining using the amalgamation technique has not been thoroughly investigated in Amansie West District. In particular, the fate of mercury in biological media is largely unknown. Therefore, research is needed to determine total Hg levels to determine the levels of mercury in hair, urine and fingernails of small–scale gold miners in the Amansie West District. The main aim of the present study was to assess levels of Hg exposure among small–scale miners actively involved in the smelting of amalgam and inhabitants in the Amansie West District of Ghana.

## Methods

Amansie West District covers a land area of about 1,141 km^2^, with Manso–Nkwanta as the capitol. The district is rich in gold deposits with mining emerging as the most vital commercial activity. It has about 310 settlements with a population of 128,862. About 52% of the population is male. Of the adult population, about 70% are farmers and 22% are engaged in artisanal mining. The district is drained in the north by the Oda and Offin rivers with Emuna, Pumpin, and Jeni as tributaries. The drainage system of the district is used for irrigational vegetable farming, cultivation of rice and to some extent aquaculture. The Offin River is well–known for gold production, and miners regularly mine using primitive methods. Several tributaries of the Offin River are dominated by artisanal mine activities. Gold is more often mined in the Offin river valley with the use of elemental liquid Hg for the amalgamation of gold particles compared to other river reservoirs in Ghana.

### Sampling

Communities in the district where small–scale gold mining activities are performed were visited. Most of the inhabitants in the Amansie West District were farmers, with young people more likely to be engaged in small–scale gold mining. The study was approved by the Ghana Environmental Protection Agency and the Ministry of Local Government and Rural Development in Manso Nkwanta. Meetings with local authorities were held to obtain their consent. Miners, including those involved in the smelting of amalgamated gold, were contacted and given information on the aims and procedures of the present study and study subjects gave their informed consent. Hair samples were cut as close as possible to the scalp following standard procedures.^28^ Samples were sealed into separate polyethylene bags. Fingernail clippings were obtained using stainless–steel clippers in agreement with International Atomic Energy Agency (IAEA) procedures to avoid to cross–contamination of specimens. For the urinary samples, miners were asked to wash their hands thoroughly to avoid any contamination. Using a 100 mL sterile plastic container, urine samples were collected in the field, sealed and kept in an ice bath. Each participant was asked to complete a questionnaire detailing their residence history, occupation, rate of fish consumption, nutritional habit, sex, and age. Hair, urine and fingernail samples were conveyed to the Department of Chemistry, Kwame Nkrumah University of Science and Technology, Kumasi, Ghana for analysis. In all, sixty–eight samples each of hair, nail, and urine were obtained from the sampling cohort. Control samples were collected from twelve individuals living in Kumasi, using the same procedure as that of the miners.

### Sample treatment and analysis

In the present study, hair samples were initially rinsed with acetone, then with deionized water and dried to constant weight in an oven at 50°C as earlier reported.^29^ Hair and nail samples were digested for THg determination using the open flask method.^30^ The weighed quantity of each sample was put into three separate boiling tubes containing 0.5 g of fingernails/hair sample, 5.0 mL of sulfuric acid, and 2.0 mL of perchloric acid: nitric acid in the ratio of 1:1. Subsequently, 1.0 mL of deionized water was added. The contents of the boiling tubes were then digested on a sand bath at 200 ± 5°C until a clear solution was obtained. The solution was cooled to room temperature and made up to the 50 mL mark with deionized water. For the urine samples, a 20.0 mL aliquot was digested with 20.0 mL sulfuric acid, perchloric acid and nitric acid in the ratio of 3:1:1. To minimize the loss of Hg by volatilization, the solution was then reduced to 20.0 mL by heating at 50°C. The digested samples were topped up to the 50 mL mark with deionized water. A standard and blank digestion using 100, 50 and 25 μL of 1μL mL^−1^ standard mercury solution was subjected to the same procedure. Samples were put in glass bottles and analyzed for their Hg concentrations using cold vapor atomic absorption spectrophotometry with an automatic mercury analyzer (HG 5000), equipped with an Hg lamp at a wavelength of 253.7 nm.

### Quality assurance

Quality assurance samples were analyzed and were comprised of replicate samples, reagent blanks and pre– and post–digestion spikes. Detection limits and recovery rate were also calculated. Samples were spiked with several concentrations of standard Hg solutions to verify the analytical procedure and recovery repeatability tests. For each batch of experiments, blanks and spiked samples were performed in triplicate using the same digestion procedure. The limit of detection was found to be 0.045 μg/L for urine samples, 0.048 μg/g for fingernail samples and 0.05 μg/g for hair samples. The recovery was greater than 90%.

### Statistical analysis

The IBM Statistical Package for the Social Sciences 20.0 software program was employed to perform the descriptive statistics. Statistical differences between mean groups were carried out using analysis of variance. The significance level was set at p < 0.05.

## Results

Table 1 lists the concentrations of Hg in the samples in the present study in non–miners (n = 12), smelter miners (n = 32) and non–smelter miners (n = 36).

**Table 1:** Concentrations of Total Mercury in Scalp Hair, Fingernails and Urine Against Duration at the Mining Site and Subject Age

Biological media	Group	Age range (years)		Duration at mining site (years)		Total mercury concentration (μg/g)		

		Range	Mean ± *SD*	Range	Mean ± *SD*	Range	Mean ± *SD*	Median
Hair	Non–miners	20–60	30 ± 0.023	–	–	0.02–2.53	0.85 ±0.001	0.41
	Smelter miners	22–56	28 ± 0.052	1–17	15 ±0.017	1.98–15.97	6.59 ± 0.006	5.36
	Non–smelter miners	19–62	24 ± 0.008	1–25	8 ±0.058	0.84–7.15	3.11 ±0.010	2.83
Fingernails	Non–miners	20–60	30 ± 0.023	–	–	0.01–0.98	0.43 ±0.001	0.39
	Smelter miners	22–56	28 ± 0.052	1–17	15 ±0.017	0.39–12.67	3.32 ±0.360	3.11
	Non–smelter miners	19–62	24 ± 0.008	1–25	8 ±0.058	0.13–7.39	2.06 ±0.001	2.32
Urine	Non–miners	20–60	30 ± 0.023	–	–	0.03–1.05	0.45 ±0.012	0.47
	Smelter miners	22–56	28 ± 0.052	1–17	15 ±0.017	2.59–12.01	6.95 ± 0.050	6.56
	Non–smelter miners	19–62	24 ± 0.008	1–25	8 ±0.058	2.04–6.14	3.35 ±0.022	2.98

Table 2 presents the Pearson correlation analysis of the concentrations of THg in scalp hair, fingernails and urine, as well as the duration of stay and age of study participants.

**Table 2:** Pearson Correlation Analysis of Total Mercury Levels in Hair, Fingernails and Urine Against Duration of Stay and Age of Smelter Miners (n = 32)

Parameters	Hair	Fingernails	Urine	Duration of stay
Fingernails	−0.244	1		
Urine	0.153	−0.012	1	
Duration of stay	0.064	−0.207	0.649^*^	1
Age	0.077	0.404	0.190	−0.361

Abbreviation:

^*^,Correlation is significant at the 0.05 level (2-tailed).

Table 3 present the one-way analysis of variance comparison of THg concentrations between and within the biological media.

**Table 3 —:** Pearson Correlation Analysis of Total Mercury Levels in Non–miners, Smelter Miners and Non–smelter Miners

	Non–miners	Smelter miners
Smelter miners	0.458	1
Non–smelter miners	0.380	0.996^*^

Abbreviation:

^*^, Correlation is significant at the 0.01 level (2-tailed).

## Discussion

As shown in Table 1, different concentrations of Hg were found in the various biomarkers of the sampled subjects. Differences were due to different levels and duration of Hg exposure, and the differing uptake of biological absorption systems. Differences in mercury levels in hair and urine could be due to burning operations at the gold mining sites.^31^ Much of the protein in hair is rich in the sulfur–containing amino acid cystine, which is largely responsible for the binding of mercury compounds. Once mercury combines with hair, it never separates and Hg levels remains consistent.^32–34^ Mercury content in hair comes from exogenous contamination and blood. Desquamated epidermis and secretions from sweat, sebaceous and apocrine glands are probably responsible for hair mercury contamination in studies.^35^

Urine Hg concentration is very stable and relatively simple, due to the characteristic of the medium.^36^ However, because organic Hg represents a very small portion of urine Hg, urine Hg is more useful for the analysis of metallic or inorganic Hg compounds.^36^ In addition, workers exposed to Hg for an extended duration exhibit high levels of urine Hg concentration for a long period of time, as seen in the blood Hg concentrations, due to the burden of Hg on the body.^37^

The Hg concentration in hair is often used as a biomarker for methylmercury exposure, as it reflects the concentrations in blood at the time the hair was formed. Simultaneously, hair sampling offers a non-invasive and simple sampling method, and as a storage method offers good sample preservation. However, hair is not as good an indicator of Hg vapor exposure as urine.^38^ The determination of toxic elements in urine is an important clinical screening procedure and has become a matter of wide interest owing to the toxicity of these elements and their influence in controlling the course of biological processes.^39^ In addition, total Hg value in urine is an important biomarker for evaluating inorganic Hg and Hg vapor exposure.^40^

The presence of Hg in urine generally represents exposure to inorganic Hg and is considered the best measure of recent exposures to inorganic Hg or elemental Hg vapors because urinary Hg is thought to indicate most closely the Hg levels present in the kidneys.^40^ Urine analysis is more useful for evaluating Hg vapor exposure of those individuals not directly involved with mining and amalgamation activities, such as employees and neighbors of gold-buying shops, as well as children and women living in mining sites. It is not surprising that smelter miners involved in open burning Hg will accumulate Hg and exhibit high Hg concentrations in urine. Therefore, in the Amansie mining communities, urinary Hg was considered to be the most valid bioindicator of exposure from inhalation of elemental vapor.

In the present study, smelter miners recorded the highest concentrations of THg in urine, hair and fingernails. However, the non–smelter miners showed greater THg concentrations than the non–miners. This was because smelter miners were either involved in operations where Hg was used or the duration of their involvement in mining activities. The presence of THg in urine, hair and fingernails of non–miners was due to Hg used in gold amalgamation processes, which volatilizes rapidly at high temperatures. This affected those living in communities close to the gold mining sites. The results presented here showed that THg in urine, hair and fingernails more efficiently distinguished mercury exposure in people close to Hg pollution sources than in people living far from the mining sites. The THg concentrations in the biological media were increased in relation to the duration of stay at the mining sites.

Similarly, Hg contamination in miners from an artisanal gold mining area in the Brazilian Amazon was higher than that in non–miners.^41^ Total mercury levels recorded in hair samples obtained from smelter miners were above the 1 μg/g limit set by the United States Environmental Protection Agency (USEPA).^42^ In contrast, only 46% and 29% of hair samples collected from non–smelter miners and non–miners, respectively, had THg concentrations above this level. The World Health Organization (WHO) considers 4 ug/L of THg in urine to be normal.^43^ Mean THg levels in the urine of smelter miners in this study were above the guideline of the WHO. Moreover, this study found that average THg concentrations measured in urine samples of non-miners and non-smelter miners were above the 4 μg/L standard. This suggests that mercury exposure identified in the smelter miners could result in mild adverse effects. The THg concentrations in this study were higher than the 0.89 – 6.50 μg/g level reported in the Pra River Basin, Ghana and 0.843 μg/g ± 0.557 in some Ghanaian individuals.^44^,^45^ The THg concentrations in the hair (4.27 μg/g), urine (6.40 μg/L) and nails (3.45 μg/g) of some artisanal miners in Tanoso, Ghana were somewhat lower than those recorded in the present study.^46^ A urine total mercury level of 1.23 ± 0.86 μg/L was reported among native individuals in Dunkwa–Offin, Ghana, a small–scale mining community.^29^

Comparing the results of the present study to those from elsewhere, a high THg concentration of 151.2 μg/g was recorded in the hair of individuals living in Brasilia Legal, Brazil and the Madeira river area (9.2 μg/g).^30^,^47^ The mean hair Hg concentration of 4.2 μg/g recorded for inhabitants of the coast of Papua New Guinea was also higher than that obtained in the present study.^48^ In the Creporizinho and São Chico areas, THg contents in urine (13.75 ± 19.59 and 17.37 ± 36.55 μg/L) among the miners were higher than those in this study, but the levels in hair (4.58 ± 2.95 and 4.50 ± 5.97 μg/g) were lower compared to the present study.^41^

In smelter miners, Hg levels in urine were found to be highest, followed by contents in hair and fingernails (*Figure 1*). Mercury levels were highest for miners around twenty–nine (29) years of age. This was because miners of this age perform more physically demanding manual tasks, such as the final stages of gold enrichment, which includes amalgam decomposition by heating and the mixing of Hg with gold–bearing rocks for Hg amalgamation.

**Figure:**
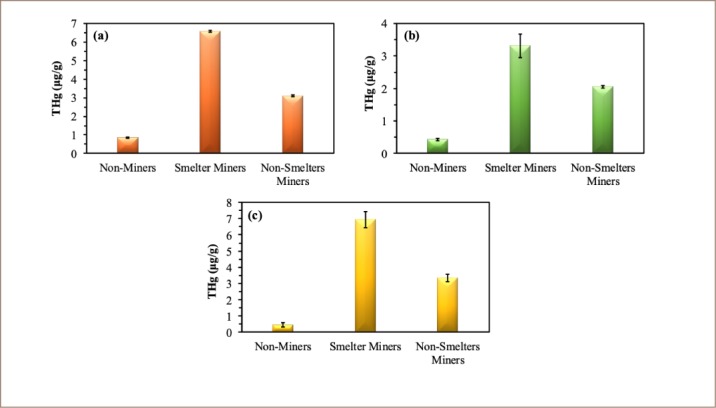
Distribution of THg in (a) hair, (b) fingernails and (c) urine of non–miners, smelter and non–smelter miners

In smelter miners, there were no differences (p > 0.05) in THg concentrations across hair, fingernail and urine samples (*Table 2*). Moreover, participant age was not associated with the amount of absorbed Hg (p > 0.05). This is in agreement with earlier studies.^43^ As seen in Table 2, older miners did not have a higher amount of Hg in hair, urine or fingernails. This may be because older miners had a shorter work duration and exposure period. Moreover, miners could have different exposures levels because of their differing work descriptions.

Pearson's correlation showed a strong significant correlation between duration of stay in mining activities and levels of THg in urine samples (r = 0.649, p < 0.05). This was comparable with earlier studies.^48^ The positive association between urine THg contents indicated that duration was a good predictor of urine Hg. However, very poor correlations between THg levels in hair (r = 0.064) and fingernail (r = −0.207) samples with respect to the duration of stay were observed. This negative correlation indicated that they were below detection limits. A weak positive correlation between THg concentrations in hair and urine of children in Germany has also been reported.^49^

The Shapiro–Wilk normality test showed that THg levels in non–miners, smelter and non–smelter miners were normally distributed (p > 0.05). Moreover, the Levene test showed homogeneous variance among study groups. The relationship among non–miners, smelter and non–smelter miners were checked by Pearson's correlation analysis. Smelter miners and non–smelter miners were significantly correlated (p < 0.01, r = 0.996), (*Table 3*). This might be due to the similar distribution behavior and mining activities of smelter and non–smelter miners. However, no significant association was observed between non–miners and smelter or non–smelter miners.

Factors such as medicines, smoking, age, alcohol consumption and place of residence were considered in the interview-administered questionnaire. None of these factors were found to influence Hg concentration in the hair, urine and fingernails of study participants. This could be due to the varied nature of the sample population with regard to background and residence in different mining sites and communities. Miners are exposed to Hg vapor at considerable rates, based on the frequent burning of amalgam in their operations.

Exposure to Hg by humans living in close proximity to mining sites primarily occurs through methylmercury from dietary sources, especially fish, and occupational Hg vapor exposure from gold melting or amalgam burning. Moreover, inhalation of Hg vapor is the primary exposure pathway for miners, gold shop workers and people living near areas where Hg is handled. Residents in the mining communities may be exposed to high methylmercury concentrations in fish from waterways that are contaminated by Hg from mining sites.

Although the use of Hg in mineral processing is illegal in most countries, including Ghana, amalgamation is the preferred method employed by ASGM practitioners. This is because Hg is inexpensive, readily available and easy to use. However, miners often ignore the health risks associated with Hg handling. Moreover, it is difficult to obtain quantitative and reliable data on Hg releases from ASGM sites, as miners do not freely offer information about the amount of Hg they use.

## Conclusions

The present study examined THg concentrations from biological media collected in the vicinity of small–scale gold mining sites in the Amansie West District. Total mercury levels measured in hair samples obtained from smelter miners were above the 1 μg/g limit set by the United States Environmental Protection Agency. In contrast, 46 and 29% of hair samples collected from non–smelter miners and non–miners had THg concentrations above this level. Moreover, the present study revealed that all of the THg concentrations measured in urine samples were far below the >25 μg/L level considered to indicate a high level of mercury contamination. This indicated low levels of THg through occupational and environmental exposure. Most of the measured THg levels were observed at higher levels in smelter miners than non–miners and non–smelter miners. The small–scale miners at the mining sites were observed handling mine tailings and Hg without the use of personal protective equipment. Based on the primary findings, factors such as migration and social connectivity, malnutrition and living downstream of ASGM activities were considered to be associated with increased mercury exposure. Based on the levels of THg analyzed in the biological media, artisanal gold mining activities in Amansie West District are on the increase with a potential risk of developing chronic effects. In conclusion, this study provides science-based exposure evidence to support the notion that individuals residing in small-scale gold mining communities are exposed to potentially high levels of Hg. In addition, it is difficult to gather accurate data on THg contamination at gold mining sites in the Amansie West District, but some of the data obtained in this study show strong evidence of Hg exposure among the smelter miners. Although individuals directly involved in gold amalgamation experienced the highest exposures to Hg, our data suggest that most of the residents were exposed to elevated levels of Hg, therefore further studies are needed to determine if such exposures are associated with adverse health outcomes. The health risks and consequences of environmental pollution must be considered and the present study will help authorities address this issue. The powers of district assemblies to monitor mining activities and impose guidelines on Hg discharges into the environment from the mining sites and to address the illegal activities of small-scale miners must be strengthened.
